# Ginsenoside Rg1 Epigenetically Modulates Smad7 Expression in Liver Fibrosis via MicroRNA-152

**DOI:** 10.1016/j.jgr.2022.12.005

**Published:** 2022-12-29

**Authors:** Rongrong Zhang, Xinmiao Li, Yuxiang Gao, Qiqi Tao, Zhichao Lang, Yating Zhan, Chunxue Li, Jianjian Zheng

**Affiliations:** Key Laboratory of Diagnosis and Treatment of Severe Hepato-Pancreatic Diseases of Zhejiang Province, The First Affiliated Hospital of Wenzhou Medical University, Wenzhou, China

**Keywords:** CCl_4_-induced fibrosis, Ginsenoside Rg1, MiRNA

## Abstract

**Background:**

Ginsenoside Rg1, a bioactive component of Ginseng, has demonstrated anti-inflammatory, anti-cancer, and hepatoprotective effects. It is known that the epithelial–mesenchymal transition (EMT) plays a key role in the activation of hepatic stellate cells (HSCs). Recently, Rg1 has been shown to reverse liver fibrosis by suppressing EMT, although the mechanism of Rg1-mediated anti-fibrosis effects is still largely unclear. Interestingly, Smad7, a negative regulator of the transforming growth factor β (TGF-β) pathway, is often methylated during liver fibrosis. Whether Smad7 methylation plays a vital role in the effects of Rg1 on liver fibrosis remains unclear.

**Methods:**

Anti-fibrosis effects were examined after Rg1 processing *in vivo* and *in vitro*. Smad7 expression, Smad7 methylation, and microRNA-152 (miR-152) levels were also analyzed.

**Results:**

Rg1 significantly reduced the liver fibrosis caused by carbon tetrachloride, and reduced collagen deposition was also observed. Rg1 also contributed to the suppression of collagenation and HSC reproduction in vitro. Rg1 caused EMT inactivation, reducing Desmin and increasing E-cadherin levels. Notably, the effect of Rg1 on HSC activation was mediated by the TGF-β pathway. Rg1 induced Smad7 expression and demethylation. The over-expression of DNA methyltransferase 1 (DNMT1) blocked the Rg1-mediated inhibition of Smad7 methylation, and miR-152 targeted DNMT1. Further experiments suggested that Rg1 repressed Smad7 methylation via miR-152-mediated DNMT1 inhibition. MiR-152 inhibition reversed the Rg1-induced promotion of Smad7 expression and demethylation. In addition, miR-152 silencing led to the inhibition of the Rg1-induced EMT inactivation.

**Conclusion:**

Rg1 inhibits HSC activation by epigenetically modulating Smad7 expression and at least by partly inhibiting EMT.

## Graphical abstract

**Figure:**
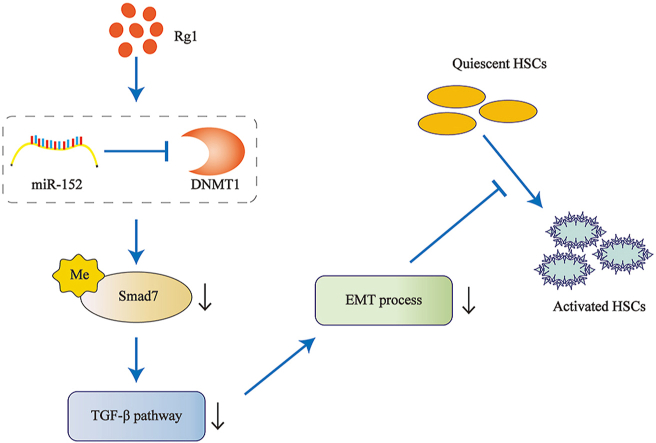

## 1 Introduction

Liver fibrosis, a reversible wound-healing reaction, is caused by a variety of chronic liver conditions such as hepatitis virus infection and by the long-term consumption of alcohol, drugs and toxins. Liver fibrosis is characterized by a lack of balance between liver extracellular matrix (ECM) production and degradation [1]. Owing to sustained liver injury, patients with persistent liver fibrosis may develop cirrhosis and even liver cancer. During liver fibrosis, hepatic stellate cells (HSCs) are activated and they transform into myofibroblast-like cells due to liver injury. HSC activation is an important source of ECM and accelerates the progression of liver fibrosis [2,3]. Thus, the effective elimination of activated HSCs is a feasible strategy for controlling liver fibrosis.

MicroRNAs (miRNAs), a class of non-coding RNA molecules with a length of 21–24 nt, bind to the 3′ untranslated region (UTR) of target messenger RNA (mRNA) to repress mRNA translation and induce its degradation [4,5]. MiRNAs function in multiple organismal processes, including cell proliferation, differentiation, and apoptosis [6,7]. miRNAs dysregulation is associated with the development of cancers and fibrotic diseases such as liver fibrosis. It is becoming increasingly clear that miRNAs, as regulators of HSC activation, play a vital role in liver fibrosis [8,9]. For example, Markovic et al demonstrated that the inhibition of miR-221 in the liver effectively prevented the progression of liver fibrosis [10]. Previously, we found that microRNA-152 (miR-152) epigenetically up-regulated Patched1 (PTCH1) expression, resulting in the suppression of the epithelial–mesenchymal transition (EMT) during liver fibrosis [11]. EMT, a process in which non-polar epithelial cells gradually transdifferentiate into mesenchymal cells, is critical for activating HSCs [12]. Therefore, the available evidence suggests that miRNAs may be potential targets for anti-fibrosis therapy.

**Fig. 1:**
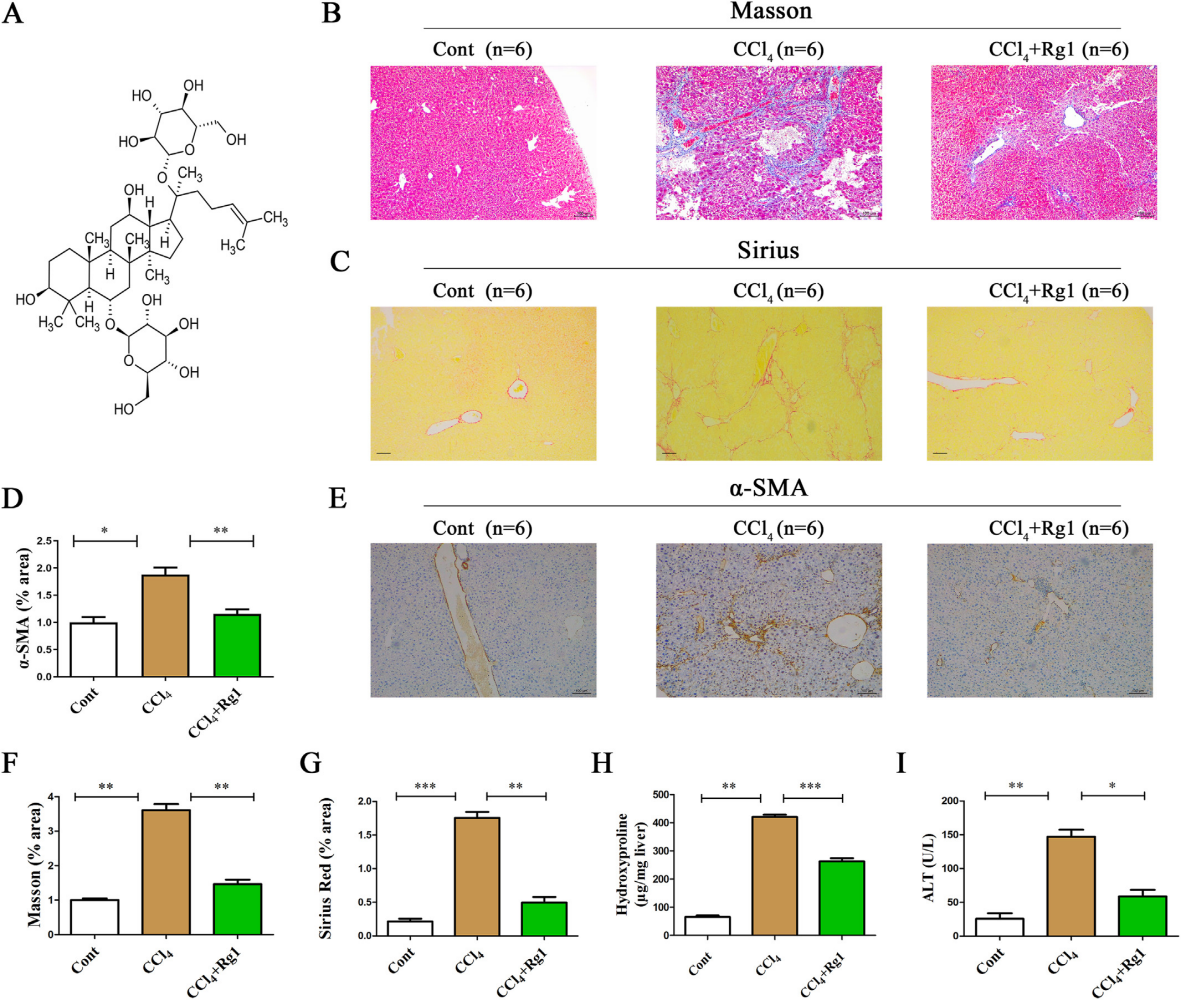
Rg1 reduces the liver fibrosis induced by CCl4. (A) Chemical structure of Rg1. Collagen deposition was evaluated using Masson (B) and Sirius red (C) staining (Scale Bar = 100 μm). (E) Immunohistochemical staining of α-SMA. The quantitative results of α-SMA (D), Masson (F) and Sirius red (G) staining after Rg1 treatment in CCl4-treated mice are shown. Hydroxyproline (H) and ALT levels (I) were analyzed after Rg1 treatment in CCl4-treated mice. ∗p < 0.05, ∗∗p < 0.01, and ∗∗∗p < 0.001.

Ginsenoside Rg1 (molecular-weight, 801.01; chemical formula, C_42_H_72_O_14_ [Fig. 1A]), one of the main bioactive components of Ginseng, has demonstrated anti-inflammatory, anti-cancer, and hepatoprotective effects [13,14]. Lu and colleagues found that Ginseng essence containing Rg1 exerts hepatoprotective effects by down-regulating oxidative stress. A recent study proved that Rg1 helps in hindering liver fibrosis [15]. Nevertheless, the mechanism of Rg1-mediated anti-fibrosis effects is still largely unclear. Interestingly, Smad7, a negative regulator of the transforming growth factor β (TGF-β) pathway, is often methylated during liver fibrosis. Whether Smad7 methylation plays a vital role in the effects of Rg1 on liver fibrosis remains unclear. Therefore, we aimed to explore the effects of Rg1 on Smad7 methylation as well as the mechanisms underlying this process.

## 2 Materials and methods

### 2.1 Materials

MiRNA-negative control (miR-NC), a miR-152 inhibitor, and a miR-152 mimic were bought from GenePharma (Shanghai, China). Carbon tetrachloride (CCl_4_) and 5-Aza-2′-deoxycytidine (5-Aza) as well as Curcumin (Cur) were obtained from Sigma (St Louis, MO, USA) and Rg1 (≥98% purity) was obtained from Abcam (Cambridge, MA, USA). Adenoviral vectors containing DNA methyltransferase 1 (Ad-DNMT1) or a contrastive scrambled sequence were obtained from GenePharma Biotechnology.

### 2.2 Animal experiments

CCl_4_ is a common hepatotoxic drug used to induce liver fibrosis [16]. To establish a mouse model of liver fibrosis, male C57BL/6J mice aged 8 weeks were intraperitoneally injected with 7 μl/g 10% CCl_4_ (Sigma-Aldrich) dissolved in olive oil twice a week for a total of 8 weeks. Eighteen mice were used and divided into three groups at random. Group 1 mice (n = 6) received intraperitoneal olive oil injections and oral PBS treatment twice per week (vehicle control); group 2 mice (n = 6) received intraperitoneal CCl_4_ injections and oral PBS treatment twice per week (CCl_4_-treated mice); and group 3 mice (n = 6) received intraperitoneal CCl_4_ injections and oral Rg1 (40 mg/kg) treatment twice per week [15]. Mice were sacrificed under anesthesia after CCl_4_ treatment. Liver tissue samples were obtained at −80°C and used for Masson and Sirius Red staining. Blood samples were collected for the analysis of alanine aminotransferase (ALT) levels using enzyme-linked immunosorbent assays (ELISAs).

The Laboratory Animal Center at Wenzhou Medical University supplied all the animals. All animal experiments were conducted after approval from the University Animal Care and Use Committee.

### 2.3 Hydroxyproline content

After being homogenized with HCl, liver tissue (50 mg) was hydrolyzed at 120°C overnight. The lysates were centrifuged at 12 000 *g* at 4°C for 10 min, and the supernatant was evaporated and dried under vacuum. The level of hydroxyproline in the liver was determined using a Hydroxyproline Colorimetric Assay Kit (BioVision). The measured values were normalized based on the weight of the liver.

### 2.4 Separation and culture of primary HSCs

Primary HSCs were separated as described previously [17]. DMEM containing 10% fetal bovine serum, penicillin (100 U/mL), and streptomycin (100 μg/mL) was used to cultivate the isolated cells. Immunocytochemical staining for α-smooth muscle actin (α-SMA) demonstrated that the cell purity was greater than 98%. Day-old primary HSCs were incubated with 50 μM Rg1, 20 μM Cur, and 2.5 μM 5-Aza alone for 24 h.

### 2.5 5-Ethynyl-2′-deoxyuridine (Edu) analyses

Rg1- and Cur-treated cells were incubated in EdU for 12 h. HSC proliferation was examined using the Cell-Light EdU Apollo 567 *in vitro* imaging kit (RiboBio) based on manufacturer's instructions.

### 2.6 Signaling pathway assay

Rg1-related signaling pathways were examined using a Cignal Finder Reporter Array (Qiagen), as described previously [18].

### 2.7 Methylation analysis

Smad7 CpG islands were detected using the UCSC Genome Browser. Sodium bisulfite-treated genomic DNA (0.5 μg) was used for PCR analysis. The Smad7 primers for PCR amplification were 5′-TCACTTGCATCTGGGGATAGC-3′ and 5′-GCCCGATTTAGACCAGCAGA-3′. To analyze the level of Smad7 methylation, bisulfite-treated DNA was analyzed as described previously [19].

### 2.8 Western blot analysis

Cells were dissociated in cracking 50 mM Tris-HCl (pH 7.4) buffer containing 2-mercaptoethanol (100 mM), SDS (2% w/v), and glycerol (10%). After separation using SDS-PAGE, proteins were electrotransferred onto PVDF membranes (Millipore, USA). The membranes were sealed in milk and incubated with primary antibodies, including anti-type I collagen, anti-E-cadherin, anti-Desmin, anti-Smad7, anti-DNMT1, and anti-β-actin (Sigma, St Louis, MO, USA) antibodies, at 4°C overnight. Subsequently, they were incubated with the secondary antibody, goat anti-rabbit IgG (1:2000, Rockland), for almost 1 h at 37°C. β-actin protein levels were used as the internal control.

### 2.9 Quantitative real-time PCR (qRT-PCR)

The Cell Total RNA Kit (Zomanbio, China) was used to extract the overall RNA from the cells. In accordance with manufacturer's instruction, 50 ng RNA was converted to cDNA using the ReverTra Ace qPCR RT kit (Toyobo). The SYBR Green master mix (Toyobo) was used for RT-PCR. The primers used for detecting *E-cadherin*, *Desmin*, *DNMT1*, and *GAPDH* levels were the same as those described earlier [11]. The primers used to detect *Smad7* expression were 5′-TTTCTCAAACCAACTGCAGGC-3′ and 5′-CCCAGGGGCCAGATAATTCG-3′. The expression level of miR-152 was examined using the Taqman MicroRNA Assay (Applied Biological system, Foster City, California). *GAPDH* levels (Applied Biological system, Foster City, California) were used to normalize the levels of other mRNAs. The relative abundance of miR-152 was normalized based on U6 snRNA levels (Applied Biological system, Foster City, California). The 2^−⊿⊿Ct^ method was used to calculate the relative gene expression.

### 2.10 Luciferase reporter assay

pmirGLO-DNMT1 was co-transfected with miR-152 or miR-NC into HEK293T cells using lipofectamine RNAiMAX-mediated gene transfer, as described previously [20]. The relative luciferase activity was normalized based on Renilla luciferase activity 48 h after transfection.

### 2.11 Statistical analysis

All date were expressed as the mean ± SD of data from three groups of separate experiments. One-way analysis of variance (ANOVA) was applied to compare variables among multiple groups. Student's t-tests were used to analyze the differences between two groups. SPSS19.0 (SPSS, USA) software was used for all analyses. P < 0.05 was considered statistically significant.

## 3 Results

### 3.1 Rg1 blocks progressive liver fibrosis *in vivo*

Masson staining was used to evaluate collagen production in CCl_4_-treated mice. As shown in Fig. 1B and F, there was greater collagen growth in mice treated with CCl_4_ than in control mice. Consistent with these results, Sirius staining revealed that collagen synthesis was higher in CCl_4_-treated mice (Fig. 1C and G). Together, these data indicated the successful establishment of a CCl_4_-induced mouse model of liver fibrosis. Interestingly, Rg1 inhibited liver fibrosis in CCl_4_ mice, resulting in reduced collagen deposition and α-SMA expression (Fig. 1B–G). Moreover, hydroxyproline analysis showed that the CCl_4_-induced enhancement in collagen levels was reversed by Rg1 treatment (Fig. 1H). Notably, the CCl_4_-induced elevation of ALT levels was attenuated by Rg1, suggesting that Rg1 contributed to the restoration of the liver after injury (Fig. 1I). Together, these data demonstrated that Rg1 contributes to the inhibition of CCl_4_-induced liver fibrosis progression *in vivo*.

### 3.2 Rg1 down-regulates HSC activation via EMT inhibition

**Fig. 2:**
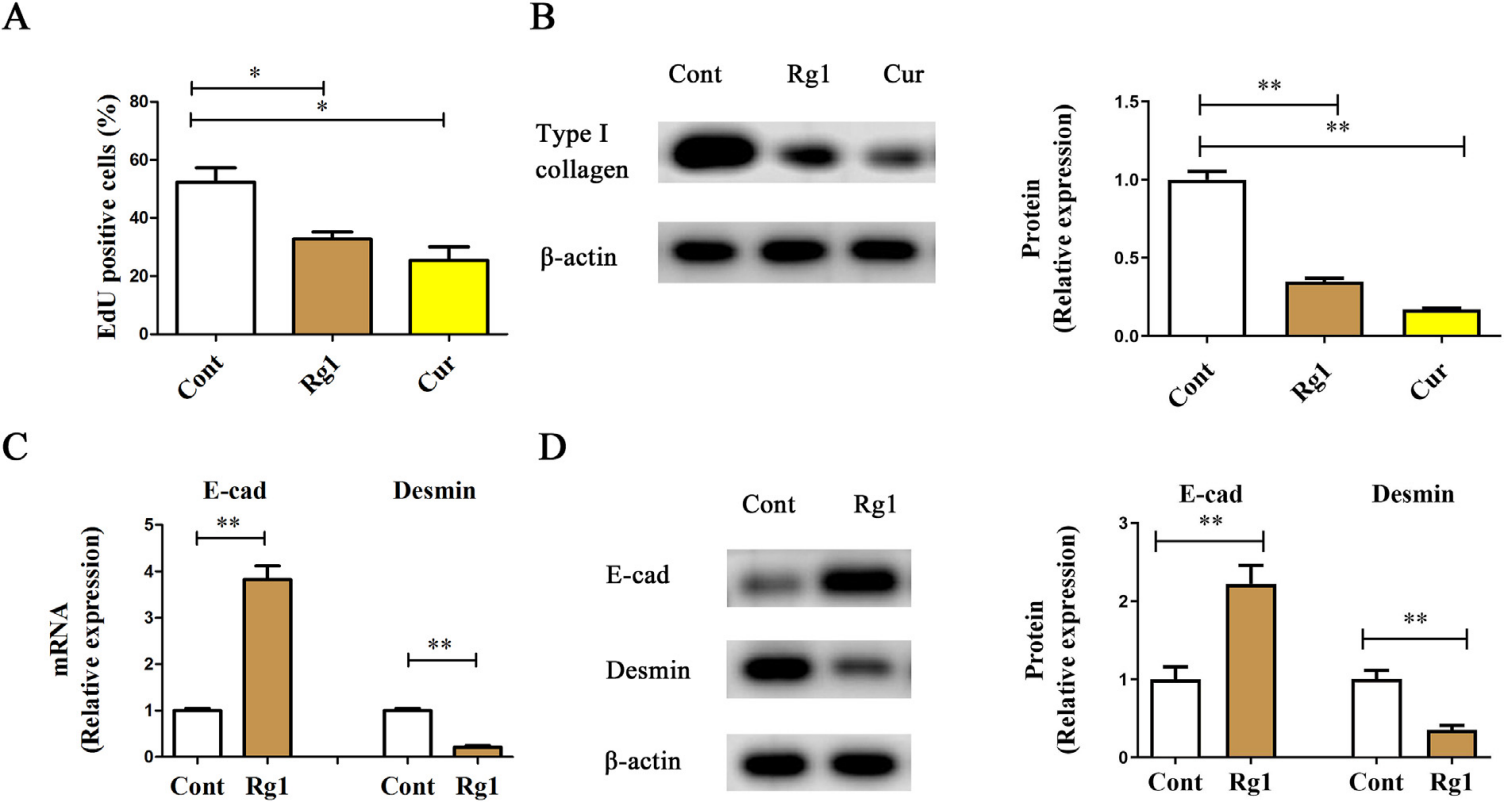
Variations in HSC activation and EMT after Rg1 treatment.Cell proliferation (A) and type I collagen levels (B) were inhibited by Rg1. The mRNA (C) and protein (D) levels of Desmin and E-cadherin were measured. ∗p < 0.05 and ∗∗p < 0.01.

In addition to *in vivo* experiments, we also examined the effect of Rg1 treatment on HSC activation. Activated HSCs are characterized by enhanced collagen expression and rapid cell proliferation. As shown in Fig. 2A, Edu analysis indicated that Rg1 inhibited HSC proliferation, similar to the results obtained after Cur treatment (positive control). Likewise, both Rg1 and Cur decreased the level of type I collagen (Fig. 2B). Next, the molecular mechanism underlying the effect of Rg1 in inhibiting liver fibrosis was explored. The mRNA levels of *E-cadherin* (epithelial marker) and *Desmin* (mesenchymal marker) were examined in HSCs treated with Rg1. Rg1 induced an increase in *E-cadherin* levels and a decrease in *Desmin* levels (Fig. 2C). Similarly, protein levels of Desmin and E-cadherin decreased and increased by Rg1, respectively, indicating the inhibitory role of Rg1 in EMT (Fig. 2D). Taken together, the data suggests that Rg1 suppresses HSC activation via the inhibition of EMT.

### 3.3 Rg1 inhibits EMT via the TGF-β pathway

**Fig. 3:**
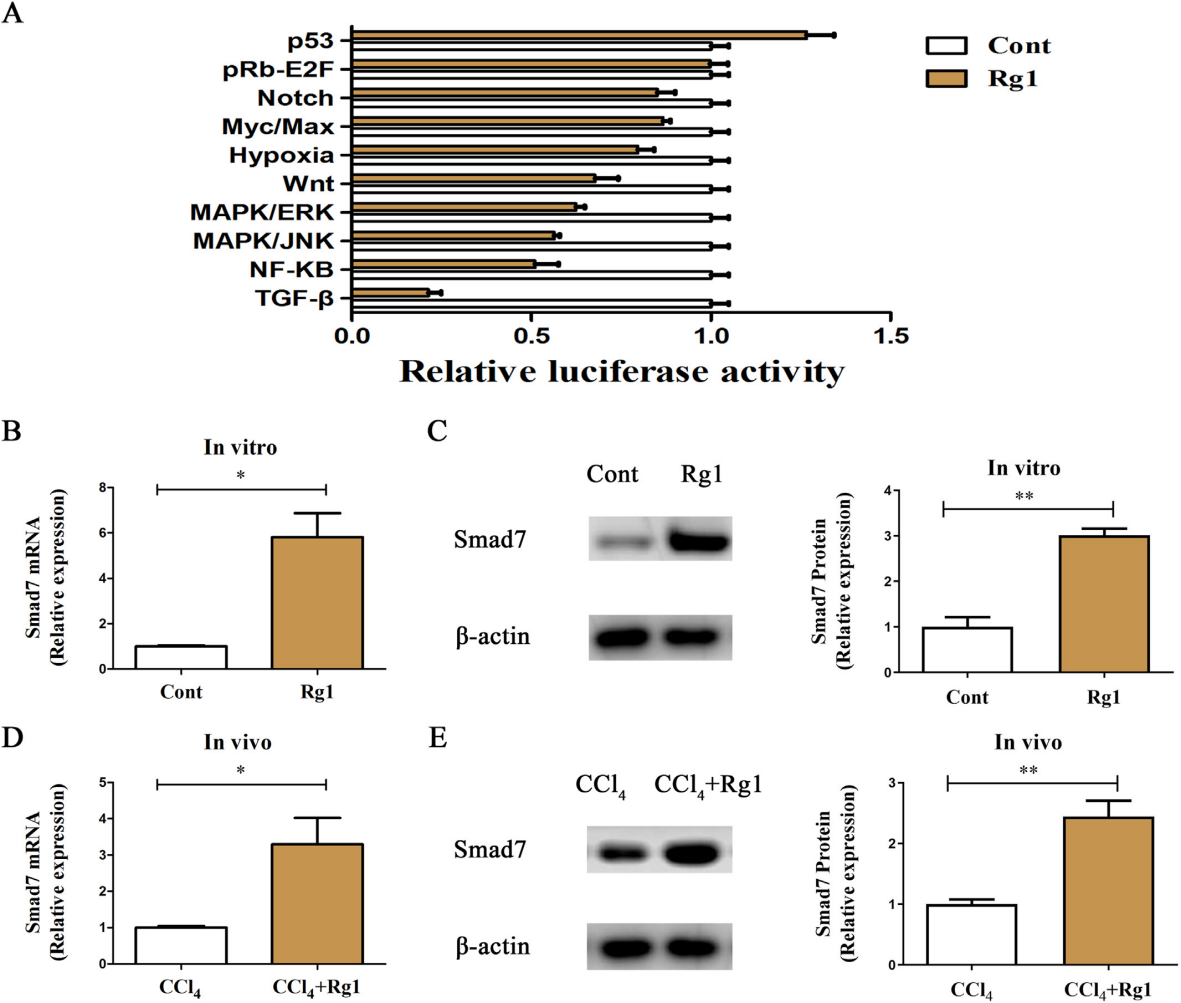
The TGF-β pathway was involved in the Rg1-mediated activation of HSCs. (A) Pathway reporter array. Smad7 mRNA (B) and protein levels (C) in vitro. Smad7 mRNA (D) and protein levels (E) in vivo. ∗p < 0.05 and ∗∗p < 0.01.

Next, we explored the Rg1-related pathways using pathway reporter arrays. As shown in Fig. 3A, most pathways were inhibited by Rg1, with the most prominent results observed for the TGF-β pathway. It is well known that the TGF-β pathway mediates EMT and promotes tumor development in cancers [21,22]. Thus, it may also mediate the effects of Rg1 on EMT. It has been reported that Smad7 is a negative regulator of the TGF-β/Smad pathway [23,24]. Therefore, we examined the mRNA and protein levels of Smad7 in HSCs treated with Rg1, both *in vivo* and *in vitro*. We found that Rg1 enhanced Smad7 expression in HSCs (Fig. 3B and C). Accordingly, Rg1 restored Smad7 levels in CCl_4_-treated mice (Fig. 3D and E). These results suggest that Rg1 promotes the inactivation of EMT via the TGF-β pathway and its negative regulator Smad7.

### 3.4 Smad7 expression is associated with DNMT1-mediated promoter methylation

**Fig. 4:**
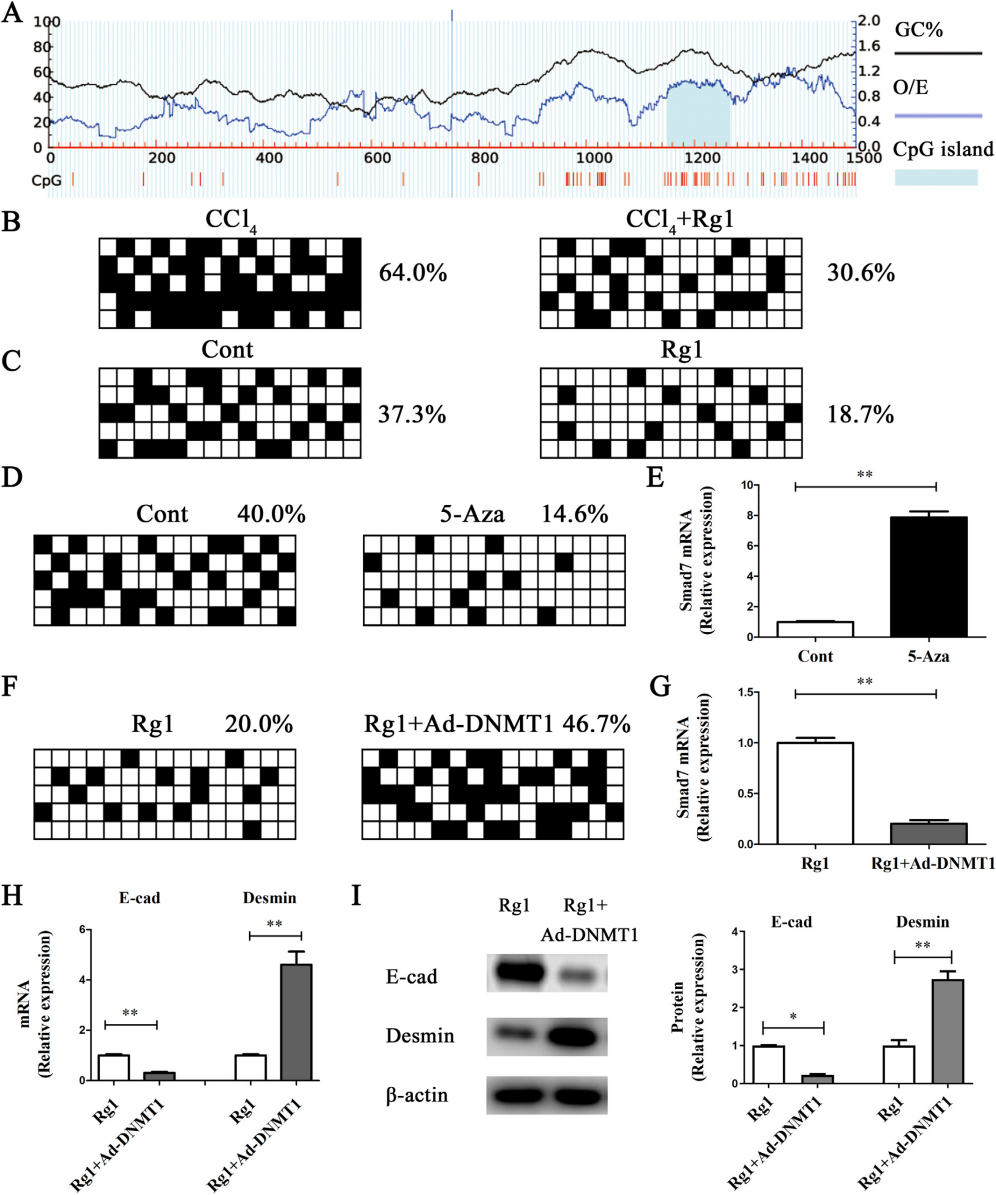
Smad7 expression was associated with Smad promoter methylation in vivo and in vitro. (A) Sketch map showing the CpG island in the Smad7 promoter region. Vertical bars represent CpG dinucleotides. The average percentage of Smad7 methylation in CCl4-treated mice after Rg1 treatment (B), Rg1-treated HSCs (C), 5-Aza-treated HSCs (D), and cells treated with DNMT1 (F). The monoclonal sequential analysis of PCR products is indicated by the lines of each box. The black box represents a methylated CpG locus while a white box represents an unmethylated locus. Smad7 content in cells after 5-Aza treatment (E) or DNMT1 overexpression (G). HSCs were treated with Rg1 for 24 h and then transduced with Ad-DNMT1 for additional 24 h. mRNA (H) and protein (I) levels of E-cadherin and Desmin. ∗p < 0.05 and ∗∗p < 0.01.

Recently, the expression of *Smad7* has been reported to be associated with its promoter methylation [25,26]. We found that the *Smad7* promoter has a CpG island with 15 CpG sites (Fig. 4A). Next, we examined whether *Smad7* promoter demethylation is involved in the Rg1-mediated inhibition of liver fibrosis. The average methylation frequency of *Smad7* in the CCl_4_ group was 64.0% (Fig. 4B). However, the CCl_4_-enhanced *Smad7* methylation was attenuated by Rg1 treatment (Fig. 4B). Accordingly, there was a reduction in *Smad7* methylation in HSCs after Rg1 treatment (Fig. 4C). Clearly, Smad7 expression *in vivo* and *in vitro* after Rg1 treatment was associated with its promoter methylation. 5-Aza, an inhibitor for DNMT, was used to treat HSCs. After treatment with 5-Aza, HSCs showed greater *Smad7* demethylation and Smad7 expression than did the control (Fig. 4D and E). Previously, Bian et al demonstrated that Smad7 levels are epigenetically regulated by DNMT1 [26]. To determine whether DNMT1 is involved in Rg1-mediated *Smad7* demethylation, the effects of DNMT1 on *Smad7* methylation and expression were examined. Overexpression of DNMT1 restored the Rg1-inhibited Smad7 methylation, and reduced Smad7 levels were observed in DNMT1-overexpressing cells even after Rg1 treatment (Fig. 4G). Further EMT-based experiments showed that DNMT1 blocked the effects of Rg1 on EMT, increasing Desmin and reducing E-cadherin levels (Fig. 4H and I). Together, these data suggest that Rg1 enhances Smad7 levels via the inhibition of DNMT1-mediated *Smad7* methylation, inactivating the TGF-β pathway as well as EMT.

### 3.5 DNMT1 is a target of miR-152

**Fig. 5:**
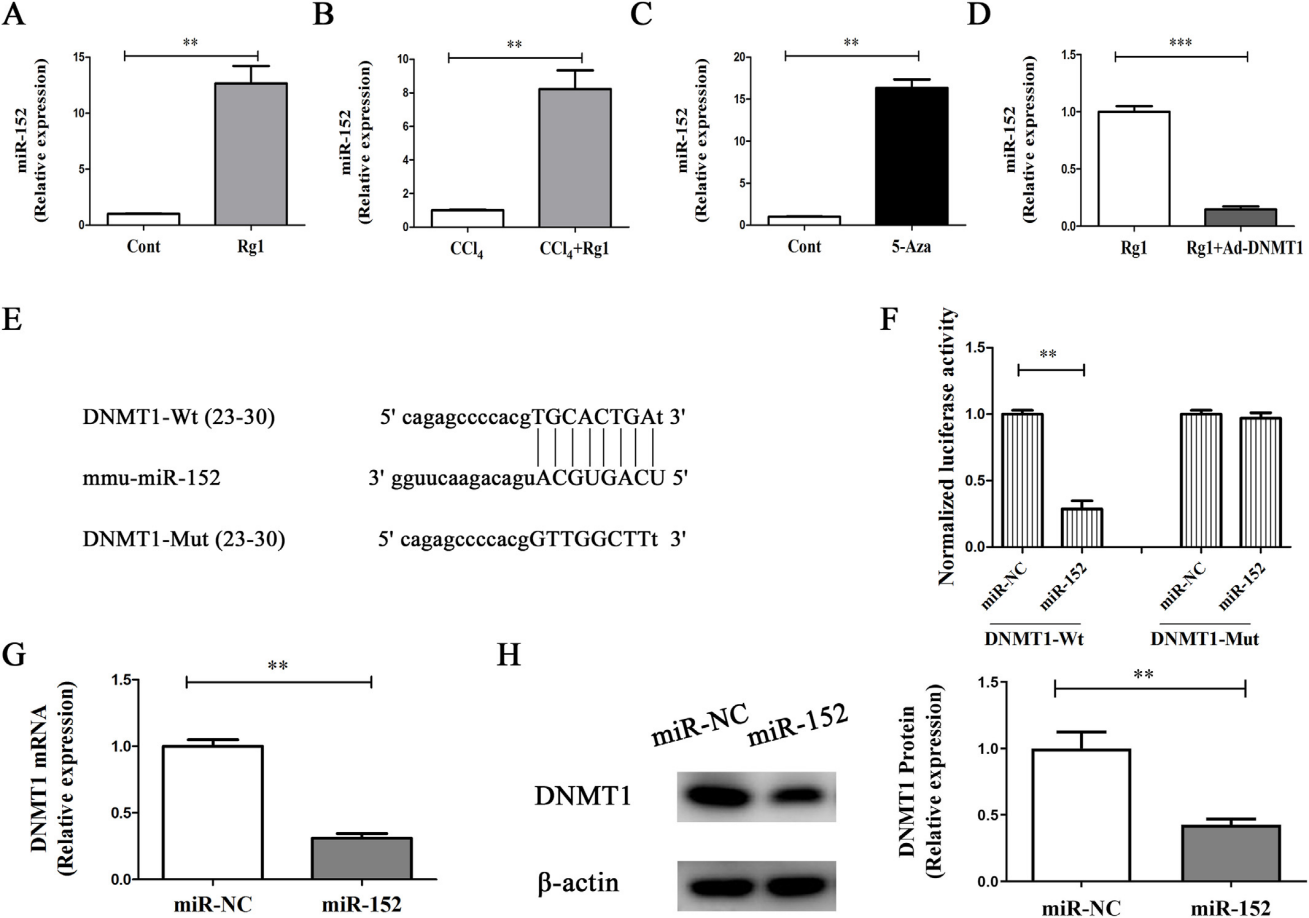
MiR-152 targets DNMT1.MiR-152 expression was examined *in vitro* (A) and *in vivo* (B) after Rg1 treatment. MiR-152 expression was examined in cells treated with 5-Aza (C) or Ad-DNMT1 (D). (E) miR-152-binding sites in the 3′-UTR of *DNMT1* mRNA. (F) Relative luciferase activity. mRNA (G) and protein (H) levels of DNMT1. Cells were transfected with 100 nM of the miR-152 mimic using lipofectamine RNAiMAX. ∗∗p < 0.01 and ∗∗∗p < 0.001.

Previously, we demonstrated that miR-152 promotes PTCH1 demethylation by inhibiting DNMT1, thereby controlling liver fibrosis [11]. We examined whether miR-152 is involved in the Rg1-mediated suppression of liver fibrosis. MiR-152 levels were elevated after Rg1 treatment, both *in vivo* and *in vitro* (Fig. 5A and B). MiR-152 levels were enhanced and reduced by 5-Aza and DNMT1, respectively (Fig. 5C and D). Bioinformatic analysis (miRDB) predicted that DNMT1 may be a target of miR-152 (Fig. 5E). Thus, DNMT1 luciferase reporters containing either the miR-152 wild-type binding site (DMNT1-Wt) or a mutated biding site (DNMT1-Mut) were generated. MiR-152 decreased the vitality of DNMT1-Wt luciferase but had no effect on DNMT1-Mut luciferase (Fig. 5F). Therefore, DNMT1 was validated as a target of miR-152. Consistent with this, miR-152 suppressed the mRNA and protein levels of DNMT1 (Fig. 5G and H). Our results suggest that miR-152, which targets DNMT1, might have an effect on liver fibrosis after Rg1 treatment.

### 3.6 MiR-152 mediates the biological effects of Rg1 on Smad7 demethylation

**Fig. 6:**
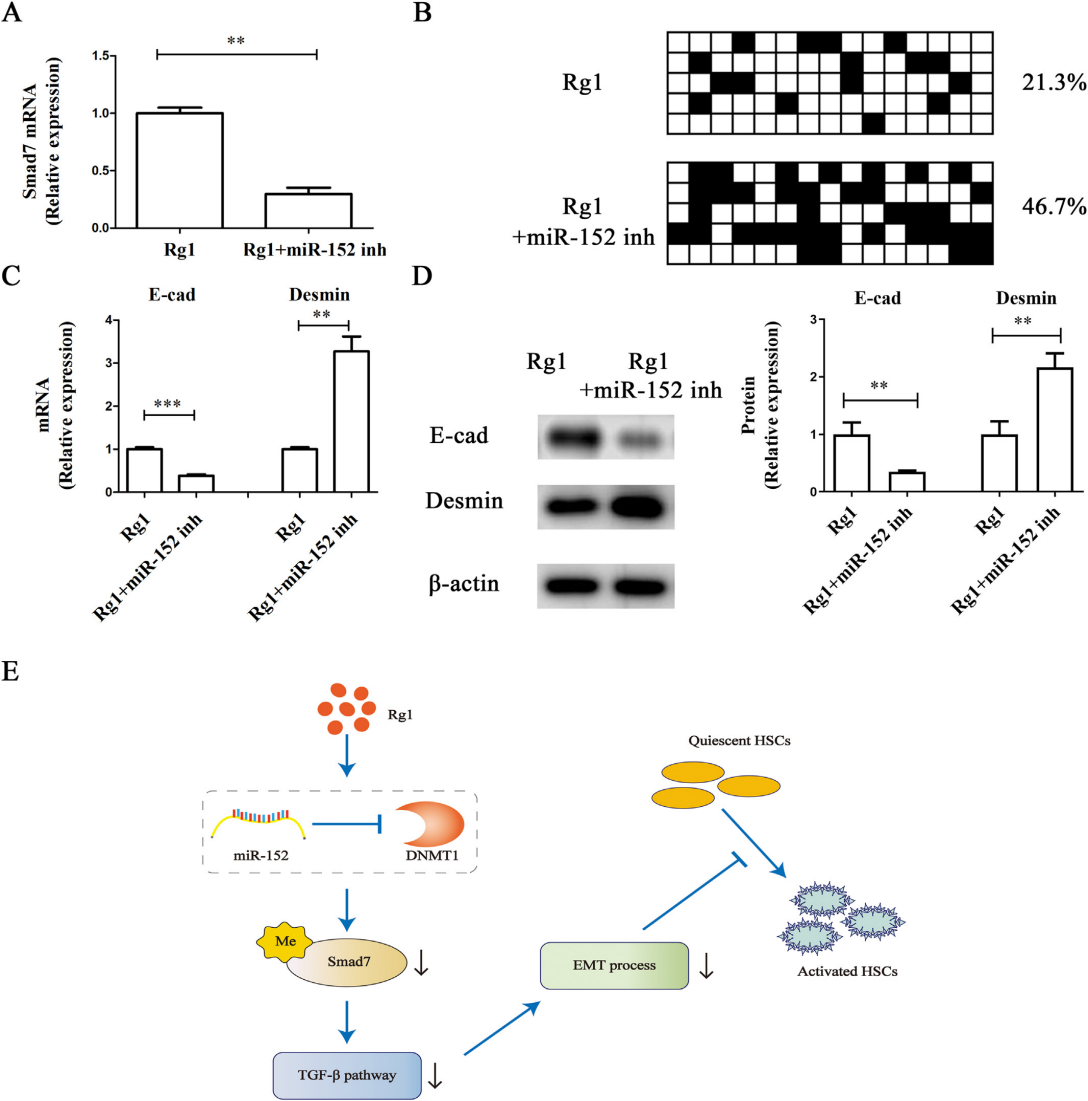
Effects of miR-152 inhibition on *Smad7* methylation and EMT. Primary 1-day-old HSCs were treated with 50 μM Rg1 and transfected with 100 nM miR-152 inhibitor for an equal time of about 24 h. (A) *Smad7* mRNA. (B) *Smad7* methylation. The mRNA (C) and protein (D) levels of E-cadherin and Desmin. (E) Schematic representation of the working model by which Rg1 epigenetically modulates Smad7 expression and contributes to EMT inactivation in activated HSCs. ∗∗p < 0.01 and ∗∗∗p < 0.001.

To determine whether miR-152 plays a vital role in Rg1-induced *Smad7* demethylation, both *Smad7* mRNA and methylation status were evaluated after miR-152 inhibitor transfection and Rg1 treatment. *Smad7* expression was markedly lower after miR-152 inhibitor transfection than after Rg1 treatment alone (Fig. 6A). Moreover, *Smad7* methylation was greater in cells transfected with the miR-152 inhibitor than in the Rg1 group (Fig. 6B). In addition, the loss of miR-152 restored Rg1-inhibited EMT, accompanied by increased Desmin and reduced E-cadherin levels (Fig. 6C and D). Therefore, Rg1 suppresses EMT in HSCs via miR-152-mediated *Smad7* methylation, at least in part.

## 4 Discussion

Rg1 has recently been reported to play an inhibitory role in a variety of human fibrotic diseases, including liver fibrosis [27,28]. For instance, Wei et al found that Rg1 inhibits liver fibrosis by inhibiting EMT as well as reactive oxygen species levels [29]. Consistent with this, the protective effects of Rg1 were confirmed in the study. Rg1 was found to inhibit liver fibrosis via EMT inactivation in our study. Subsequently, increased Smad7 expression and enhanced *Smad7* demethylation were found in Rg1-treated HSCs. Interestingly, miR-152-mediated DNMT1 inhibition was found to be responsible for *Smad7* methylation in Rg1-treated cells. Finally, we revealed the involvement of miR-152-mediated *Smad7* demethylation in the anti-fibrotic mechanisms of Rg1 (Fig. 6E). To our knowledge, this is the first report of such a finding.

EMT, a highly conserved evolutionary process, is involved in various human diseases including chronic inflammation, cancer, and fibrotic diseases [30,31]. Aberrant EMT promotes HSC activation [32]. Increasing evidence shows that TGF-β, a strong inducer of EMT, facilitates the progression of cancers as well as fibrotic diseases [33,34]. Similarly, our study showed that the TGF-β pathway is involved in the Rg1-mediated inhibition of HSC activation, with an increase in Smad7 expression and a reduction in *Smad7* methylation. *Smad7*, inhibiting the TGF-β pathway, was methylated and down-regulated during HSC activation, and this effect was reversed by Rg1. Further examination revealed that 5-Aza, a DNMT inhibitor, also promoted the demethylation of *Smad7* and the restoration of Smad7 expression, suggesting the involvement of epigenetic regulation in the anti-fibrotic effects of Rg1. Taken together, these results suggest that Rg1 inhibits EMT and thus inhibits HSC activation, at least in part via the epigenetic modulation of Smad7 expression.

DNA methylation, a common epigenetic modification, is often associated with disease progression and is involved in the regulation of gene transcription [35]. New studies have demonstrated that DNA methylation may accelerate liver fibrosis along with HSC activation [36]. For example, the hypermethylation of *PTEN* caused by DNMT1 has been reported to down-regulate PTEN expression, leading to the enhancement of HSC activation [37]. Bian et al found that DNMT1, responsible for the maintenance of methylation, contributes to *Smad7* methylation in a rat liver fibrosis model [26]. Herein, our results revealed that DNMT1 overexpression significantly inhibits the Rg1-induced *Smad7* demethylation, and causes a decrease on Smad7 expression. DNMT1 also blocks Rg1-induced EMT inactivation. Therefore, DNMT1-mediated *Smad7* methylation inhibits the effects of Rg1 on EMT and HSC activation.

MiR-152, a member of the miR-148/152 family, is often down-regulated in some human diseases such as cancers [38]. MiR-152 has been reported to act as a tumor suppressor in a variety of cancers [39]. For example, miR-152 overexpression suppresses colorectal cancer progression by down-regulating AKT and ERK pathways [38]. Recently, aberrant miR-152 expression was observed in liver fibrosis. Li et al demonstrated that a reduction in miR-152 accelerates liver fibrosis [40]. In a previous study, the DNMT1-mediated hypermethylation of *PTCH1* was shown to be blocked by miR-152 [11]. In the present study, miR-152 levels were up-regulated both *in vivo* and *in vitro* after Rg1 treatment. In Rg1-treated HSCs, DNMT1 overexpression led to the down-regulation of miR-152, whereas 5-Aza treatment induced miR-152 expression. DNMT1 was validated as a miR-152 target through a luciferase assay. In addition, miR-152 silencing was found to suppress Smad7 levels and enhance *Smad7* methylation. Notably, the loss of miR-152 restored the activation of EMT in Rg1-treated cells. Therefore, our data demonstrated that Rg1 impedes EMT in HSCs via miR-152-mediated *Smad7* demethylation. The detailed mechanism of the regulation of Rg1 in miR-152 is still unknown, and further studies are needed in future.

Together, our study shows that Rg1 up-regulates miR-152, leading to the demethylation of *Smad7* and a restoration of its expression, which causes an inhibition of the TGF-β pathway and EMT in HSCs. These results provide new insights into the antifibrotic mechanisms underlying the effects of Rg1 in treating liver fibrosis.

## Declaration of competing interest

The authors declare that they have no competing interests.
