# Supplementary material for: Ginsenoside Rg1 Epigenetically Modulates Smad7 Expression in Liver Fibrosis via MicroRNA-152
Source: J Ginseng Res. 2022 Dec 29;47(4):534–42. doi: 10.1016/j.jgr.2022.12.005 (PMC10310870; doi:10.1016/j.jgr.2022.12.005)

**Supplementary Material**

**Fig.S1** Effects of different concentrations of Rg1 on HSC activation. (A) Cell proliferation. (B) EMT markers. * p < 0.05 and ** p < 0.01.


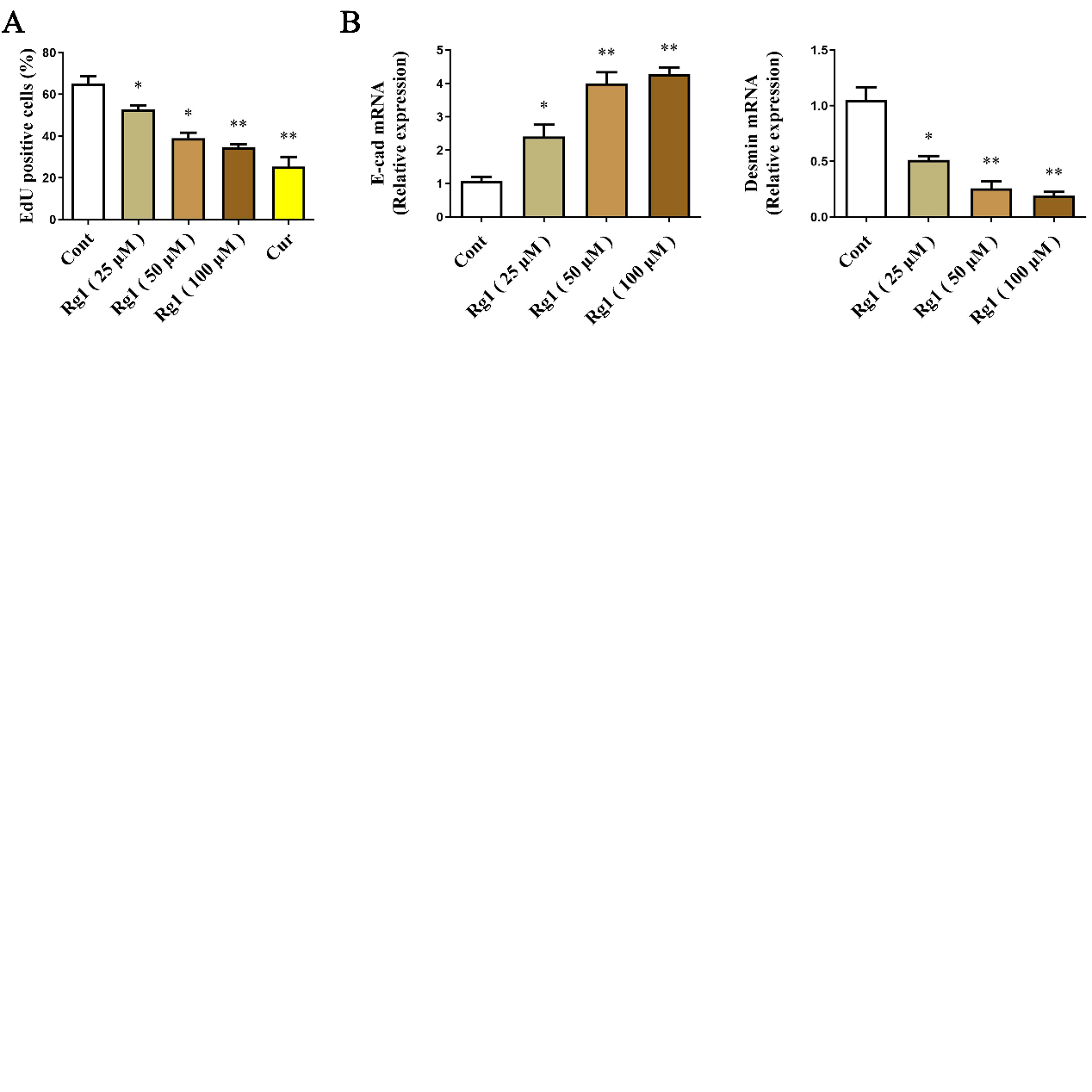


**Fig.S2** Smad7 levels in cells with different concentrations of Rg1. (A) Smad7 RNA expression level. * p < 0.05 and ** p < 0.01.


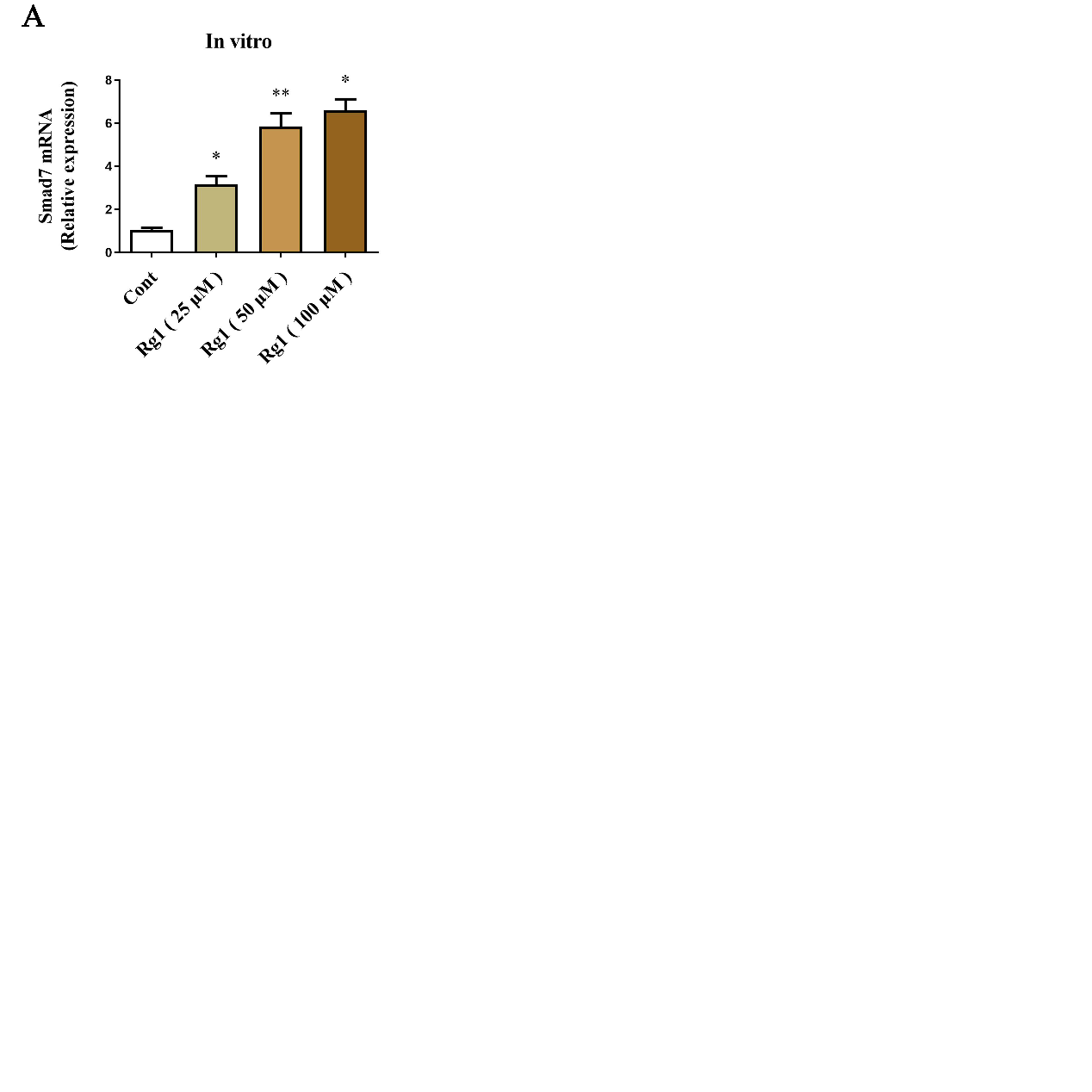

Supplement: Multimedia component 1 [file mmc1.docx]
